# Lung ultrasound education: simulation and hands-on

**DOI:** 10.1259/bjr.20200755

**Published:** 2021-03-02

**Authors:** Stephen Wolstenhulme, James Ross McLaughlan

**Affiliations:** 1 Department of Radiology, Leeds Teaching Hospitals NHS Trust, Leeds, UK; 2 School of Electronic and Electrical Engineering, University of Leeds, Leeds, UK; 3 Leeds Institute of Medical Research, St James’s University Hospital, University of Leeds, Leeds, UK

## Abstract

COVID-19 can cause damage to the lung, which can result in progressive respiratory failure and potential death. Chest radiography and CT are the imaging tools used to diagnose and monitor patients with COVID-19. Lung ultrasound (LUS) during COVID-19 is being used in some areas to aid decision-making and improve patient care. However, its increased use could help improve existing practice for patients with suspected COVID-19, or other lung disease. A limitation of LUS is that it requires practitioners with sufficient competence to ensure timely, safe, and diagnostic clinical/imaging assessments. This commentary discusses the role and governance of LUS during and beyond the COVID-19 pandemic, and how increased education and training in this discipline can be undertaken given the restrictions in imaging highly infectious patients. The use of simulation, although numerical methods or dedicated scan trainers, and machine learning algorithms could further improve the accuracy of LUS, whilst helping to reduce its learning curve for greater uptake in clinical practice.

## Introduction

COVID-19 can cause massive damage to the lung alveoli, which can result in progressive respiratory failure and potential death.^1^ Chest radiography and CT are the primary imaging tools used to aid the diagnose and monitor COVID-19.^2^ These modalities have a range of limitations such as the use of ionizing radiation; and the potential movement of the critically ill, patients to/from the imaging department, which may increase patients’ and staff radiation exposure and infection risk.^2–4^

Before and during the COVID-19 pandemic, point-of-care (POC) ultrasound (US, POCUS) has been developing.^4–8^ Lung ultrasound (LUS) during COVID-19 is being used to aid decision-making and improve patient care: the stratification of lung disease; and monitor disease progression, or resolution after treatment^4,6,7^ It has limitations that require addressing before it’s widespread implementation during and beyond the pandemic:

- Limited non-radiology and radiology practitioners with the required skill mix to do the examination,^2,4,6,7^
- Where and when to do examinations,^5–7^
- Potential infection risk for the practitioner and other humans,^3,4,6,7^
- Anecdotally reduced diagnostic confidence when wearing personal protective equipment (PPE),
- Reproducibility of the technique and image interpretation,^6,8^
- Governance – education, the examination, continuing professional development and quality assurance (QA).^4^

This article will explore, for patients with suspected COVID-19, or other lung disease, the role and governance of LUS.

### Who should be doing the LUS examination?

During the early phases of COVID-19, LUS is being done by non-radiology practitioners such as emergency medicine physicians, intensivists, nephrologists, obstetricians, paediatricians, and physiotherapists^6,7^, and radiology practitioners (radiologists, and sonographers).^2,4^ To improve patient care, we believe LUS should be a core competence for all non-radiology and radiology practitioners who are involved with the diagnosis, treatment, and monitoring of patients with suspected and/or proven lung disease. They should be qualified/certified to perform LUS. To achieve this, research is needed to determine the skill mix and level of expertise required to deliver best practice.

### Where and when should LUS be done?

Critically ill adult and pediatric patients in resource poor/medium/rich settings with suspected lung disease should have POC LUS.^4–7^ The locations include: (a) primary care – the family (general) practitioner’s surgery and a patient’s home/nursing home^6^; and (b) secondary care – emergency medicine departments, the intensive care unit, paediatric-/adult- medical/surgical wards, and obstetric wards.^6,7^ This to: (a) diagnose conditions such as consolidation, COVID-19, interstitial lung disease (ILD), pleural effusions and respiratory distress syndrome (RDS)^5^; b) guide therapeutic intervention,^4,5^ and (c) monitoring of disease progression or resolution^4,6,7^ The ideal time to do LUS is when the patient needs it,^5–7^ and could be done 24 h of the day, seven days a week^9^ However, research is needed to determine the skill mix and level of expertise required to deliver this practice.

### Which US equipment to use?

**Figure 1.**
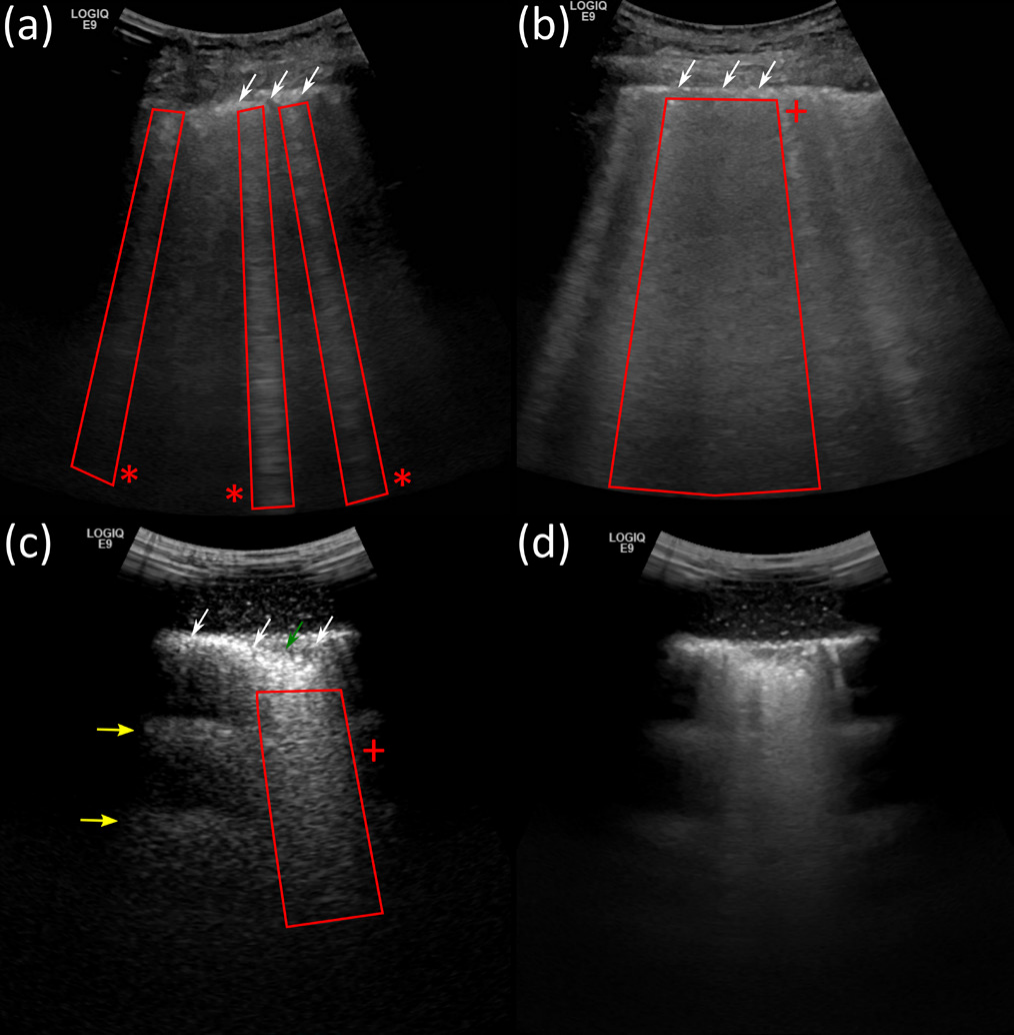
Shows images acquired using a GE E9 with a C 1–6 MHz probe. (a) A lung ultrasound (LUS) scan of an adult COVID-19 patient with a thickened irregular pleural line (white arrow) and b-lines (red asterisk box), (b) LUS of an adult COVID-19 patient with b-lines, and coalescent b-lines “white lung”(red +box), (c) and d) LUS phantom images with a-lines (yellow arrows), coalescent b-lines “white lung”(red +box) and a small sub pleural consolidation (green arrow). (c) is without advanced image processing (compound -(CI), speckle reduction- (SRI), tissue harmonic imaging (THI)) and low dynamic range (DR) 57), and d) with advanced image processing (the highest-level of CI and SRI, THI and high DR 72).

**Figure 2.**
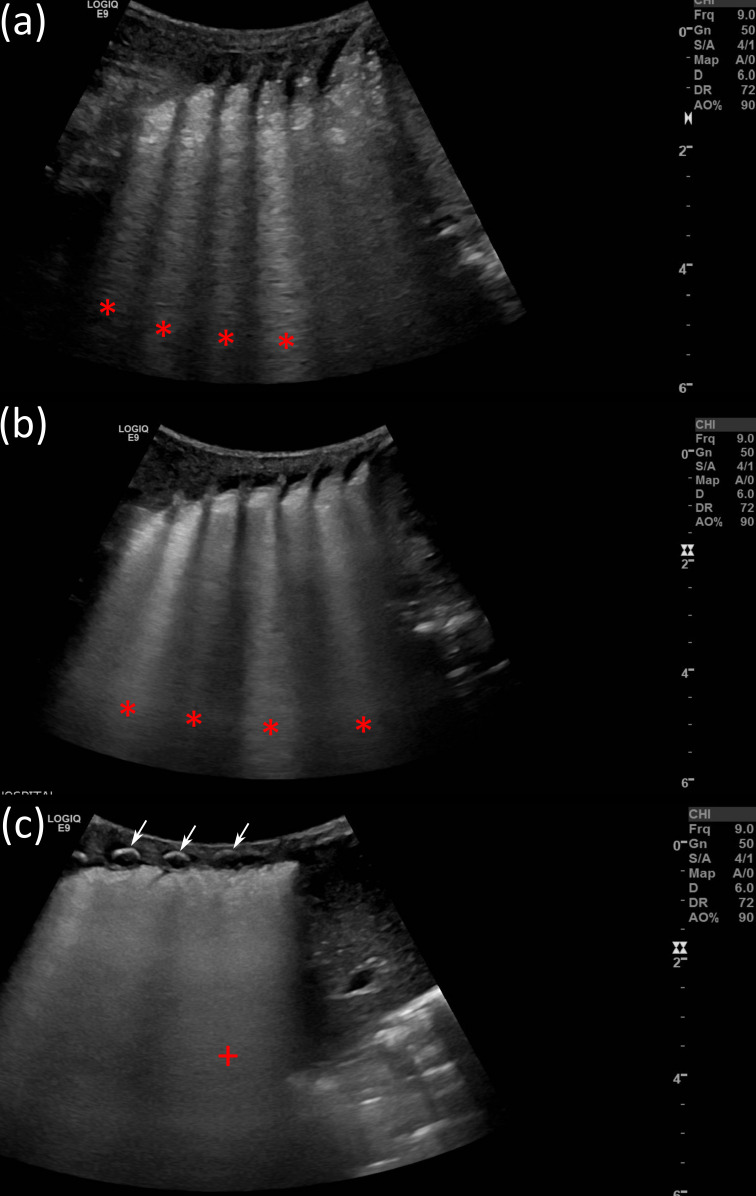
Shows images of a neonatal LUS acquired using a GE S7 with a C 2–9 MHz probe. (a) and b) show b-lines (red asterisks) without (a) and with (b) advanced image processing. (c) neonatal LUS showing ribs (white arrows), a thickened pleural line, and coalescent b-lines ‘white lung’ (red +) with advanced image processing and a high dynamic range.

For patients with suspected COVID-19, or other lung disease, POCUS is convenient, due to its: low cost, dynamic examination, non-ionising radiation, and portability.^5,6,10,11^ The US scanner should:

- Be handheld, or a cart and easy to clean,^4,11^
- At minimum have a curvi-linear, low-frequency (Figures 1 and 2) and a linear, high-frequency probe^4–8^
- Potentially have advanced image processing (AIP): compound-, harmonic-, speckle reduction- and color Doppler imaging.^10^

This allows scanning: (1) in confined bed spaces with limited access to a power supply; (2) using best practice infection prevention control^4,6,7^; (3) using the traditional technique without AIP, (Figure 2c)^4,5^ or with AIP (Figures 1 and 2). AIP can be used to improve the signal-noise ratio^10^ with the potential, of better demonstration of pleural line thickening, subpleural consolidation, b-lines and confluent b-lines (white-lung) (Figure 1), which are used to aid the stratification and monitoring of COVID-19.^6,7^ Research is needed to evaluate in COVID-19 disease LUS with AIP compared to the traditional technique^4–7^ against diagnosis with a CT scan and patient outcome.

### Education

LUS is not an easy examination, it requires expertise in pattern recognition and has a steep learning curve.^4–8^ Practitioners who learn LUS can be undergraduates and postgraduates. The practitioner should not be a complete novice; one with no skills or knowledge of US physics and application techniques. Moving the probe across the regions of the chest^5^ and interpreting subtle pathology associated with COVID-19,^6,7^ and other lung disease^5^ requires skills that cannot be obtained in a short period.^4–6,8^ LUS, unlike most soft tissue imaging with US, relies on the observation and interpretation of US artifacts.^4–8^ The practitioner needs to be able to interpret normal and abnormal appearances to detect lung disease. Covid-19, ILD and RDS present on LUS as b-lines.^5,6^ These are discrete vertical hyperechoic reverberation artifacts, which originate at the pleural line and often extend through to the maximum imaging depth, as illustrated in Figures 1 and 2.^4–6^ They move with respiration and up to three can be present in healthy lung.^5^ The observation of three or more is an indication of lung pathology.^4–7^ In severe cases, coalescent b-lines filling the space between ribs (white lung) are detected^5–7^ (Figures 1 and 2).

Historically, practitioner LUS education would be didactic teaching and hands-on training using US exploration of normal people and patients with a variety of pathologies, then passage to Covid-19 patients, as usually occurs for other US examinations. The Covid-19 pandemic poses several problems for educators, clinical practitioners and ultimately the patient:

- The rapidly emerging pathology and its high infective potential;
- The increasing number of patients admitted to hospital with COVID-19;
- The backlog of non-COVID patients on radiology and non-radiology departments;
- The high number of practitioners who need to master the technique in a short period of time.

During the COVID-19 pandemic and beyond, therefore, to, quickly and safely, enhance the practitioner’s learning and develop a high level of knowledge and skills of the LUS technique and image interpretation, different types of education will be required:

- Didactic lectures. The didactic lectures should be delivered online prior to the face-to-face sessions.^2,4,8^ They should include: an introduction to US physics, LUS artefacts, technique, image interpretation and reporting, diagnostic accuracy, reproducibility, and pitfalls.^4,6,8^
- *In vitro* Simulation – Lung phantoms. Practitioners should initially gain knowledge and skills by simulation^12^ for the interpretation of LUS artefacts using an US scanner and a low fidelity lung phantom.^13^ (Figures 1 and 2) Then advance to using web-based learning^2,4,8^ and high-fidelity simulations systems with haptic feedback with images produced from patient data.^4^
- *In situ* Simulation – Healthy volunteers. The practitioner should practice the LUS technique and interpretation of normal anatomy and artifacts^4,6–8^ on a healthy volunteer.^12^ This to develop mastery before scanning a patient.^8,12^
- Radiology department. LUS education within the radiology department will be limited due to social distancing, and increased scan times, because of wearing PPE, to reduce the spread of COVID-19.^2,4,6^
- Point of care. It is suggested the novice will require a minimum of 25 supervised LUS examinations and an assessment to be deemed competent.^8^ This will be a challenge due to the limited number of LUS POCUS trainers; and the risk of transfer of COVID-19, from the patient to the novice, and the expert trainer, in a confined POC space,^6^ Research of novel ways of POC LUS education need to be considered.
- Certification of competency. The core competency for all non-radiology and radiology practitioners doing LUS could be assessed using the international multispecialty consensus tool: the *Objective Structured Assessment of Ultrasound Skills* (OSAUS).^14^ This comprises of seven elements describing essential sub steps of an US examination. It enables assessment of practitioner performance through training until competency rather than relying on a predetermined number of scans. Using the OSAUS generic rating scale, during training for assessment, with structured formative feedback has the potential to improve the practitioner’s LUS skills acquisition. Research is needed to determine the effectiveness of using the OSAUS tool for the LUS setting.

### Quality assurance

POCUS in acute and critical care settings undertaken by non-radiology and radiology practitioners needs the same governance. The indication for LUS needs to be justified; and the images, and a typed report, should be stored in a secure location such as a hospital’s or national healthcare system’s picture archiving and communication system (PACS). This to allow the LUS examination to be part of the patient’s record, which can be reviewed at any time by secondary care, non-radiology, and radiology practitioners, to aid rapid and future patient management.^4^ The images and reports need regular peer-review, and discussion at local education meetings, to ensure quality assurance and the practitioner’s continuing professional development.^4^

### The future

A need for simulation of US imaging, in freely available US software packages, such as k-Wave,^15^ to allow for experimentation in as wide as possible parameter space is needed. This to provide the opportunity for practitioners and medical physicists to refine image settings and probe choice without patient exposure.^10^ Furthermore, as the use of machine learning to aid in the diagnosis of LUS patients increases, it will help reduce the learning curve for the effective use of this modality.^16^

The logistics of setting up a LUS service during and beyond the COVID-19 pandemic will be challenging. To become best care, evidence-based practice, this needs collaboration between the non-radiology and radiology departments, Higher Education Institutions, and US scanner and LUS phantom manufacturers.

## Conclusion

For patients with suspected COVID-19, or other lung disease, the role of POC LUS is developing. It requires practitioners with relevant education to ensure timely, safe, and diagnostic clinical/imaging assessments for rapid decision-making.^8^ Education using simulation and hands-on will enhance learning and improve patient care.^12^ The aim is with good governance, LUS will be best practice in the patient pathway for COVID-19 and beyond.
